# Geographical classifications to guide rural health policy in Australia

**DOI:** 10.1186/1743-8462-6-28

**Published:** 2009-12-08

**Authors:** Matthew R McGrail, John S Humphreys

**Affiliations:** 1 Gippsland Medical School, Monash University, Northways Road, Churchill, Victoria, 3842, Australia; 2 School of Rural Health, Monash University, PO Box 666, Bendigo Central, Victoria, 3552, Australia

## Abstract

The Australian Government's recent decision to replace the Rural Remote and Metropolitan Area (RRMA) classification with the Australian Standard Geographical Classification - Remoteness Areas (ASGC-RA) system highlights the ongoing significance of geographical classifications for rural health policy, particularly in relation to improving the rural health workforce supply. None of the existing classifications, including the government's preferred choice, were designed specifically to guide health resource allocation, and all exhibit strong weaknesses when applied as such. Continuing reliance on these classifications as policy tools will continue to result in inappropriate health program resource distribution. Purely 'geographical' classifications alone cannot capture all relevant aspects of rural health service provision within a single measure. Moreover, because many subjective decisions (such as the choice of algorithm and breakdown of groupings) influence a classification's impact and acceptance from its users, policy-makers need to specify explicitly the purpose and role of their different programs as the basis for developing and implementing appropriate decision tools such as 'rural-urban' classifications. Failure to do so will continue to limit the effectiveness that current rural health support and incentive programs can have in achieving their objective of improving the provision of health care services to rural populations though affirmative action programs.

## Introduction

"From 1 July 2009, the outdated and flawed Rural, Remote and Metropolitan Areas (RRMA) system will be replaced by the Australian Standard Geographical Classification - Remoteness Areas (ASGC-RA) system" [1]. The ongoing significance of geographical classification schema as the basis for significant health resource allocation was highlighted again with the above announcement in the Australian Government 2009 Budget. The implications and impacts of these changes have already been noted in the media [2-6], with health practitioners, organisations and professional associations immediately expressing concern about potential loss of income as a result of no longer qualifying for additional incentive payments associated with degree of rurality and remoteness. In addition to the direct impact, the indirect effects of income supplementation in attracting health workforce to areas that are traditionally difficult to recruit and retain have major repercussions for residents and services in these areas. Although there is no 'natural' classification of what constitutes 'rural' or 'remote', it is recognised that the way in which populations and communities are delimited as urban, rural and remote has important implications for health care planning and policy. Rural Australia, which contains approximately one-third of the population (ASGC-RA, excluding Major Cities), is extremely heterogeneous, comprising vast regions of sparsely populated and mostly uninhabitable areas along with small isolated rural towns and larger regional centres. Vast distances separating many of these localities, often in combination with their small population base, mean that the delivery of health care services to most of rural and remote Australia requires funding assistance through the allocation of resources to compensate for disadvantages associated with geography [7]. Decisions underpinning the distribution of these resources need to be made using geographical classification formulae. Since this issue was discussed a decade ago [8], significant policy changes have occurred that have only served to heighten the significance of how different classifications, which form the basis for resource funding, are determined. The aim of this paper is to show why geographical classifications have such an important influence for workforce recruitment and retention policies and incentives in non-metropolitan areas. In particular, this paper critically reviews the design of current geographical classifications used in Australia and their appropriateness as the basis for rural health workforce policy and resource allocation.

## Current geographical classifications used in Australia

Australia has always been a key player in the development of geographical classifications designed to capture or measure comparative degrees of rurality and remoteness (see for example Lonsdale & Holmes [9]; Logan et al. [10]). Three classifications, the Rural, Remote and Metropolitan Area (RRMA), the Accessibility/Remoteness Index of Australia (ARIA) and the Australian Standard Geographical Classification Remoteness Areas (ASGC-RA, originating from ARIA), have dominated recent rural health policy in Australia:

i. The RRMA classification had its origins in the Department of Primary Industries and Energy and the Department of Community Services and Health, and was released in 1994 [11]. This classification divides all Statistical Local Areas (SLAs) of Australia into three zones, namely metropolitan, rural and remote and a total of seven categories across these zones. The separation of rural and remote zones is determined using a method earlier developed by Arundell [12], by weighting five indicators that measure population density and straight - line distances to various population centres. Significantly, after the identification of remote areas, separation into the seven categories of rurality was determined solely based on the size of the largest population centre within each SLA.

ii. The ARIA classification, developed by GISCA, was released in 1999 [13]. Unlike RRMA, ARIA is not restricted to using pre - defined spatial units (e.g. SLAs) by utilising a one kilometre grid that covers all of Australia. The ARIA classification, intentionally designed to measure geographical remoteness, is calculated using road distances separating localities from four levels of service centres distinguished by population size. The final ARIA score is determined by aggregating these four measures of remoteness, which are then separated into five hierarchical ('natural break') categories.

iii. In 2001, the Australian Bureau of Statistics (ABS) adopted a slightly altered methodology, referred to as ARIA+ [14,15], with one key difference being the addition of a fifth service centre level. From this, a new classification known as ASGC-RA superseded ARIA. Additionally, ASGC-RA adopted a different set of hierarchical categories, with five defined again but utilising a different range of scores and a different set of category labels.

Table 1 summarises some of the strengths and weaknesses identified within these three classifications [15-17]. The key strength of the ARIA and ASGC-RA classifications is that they were designed to directly address all the weaknesses of RRMA by improving the flexibility, precision, stability and clearer conceptualisation [15,18]. However, until now, RRMA remains a key classification within rural health policy, with many specific purpose programs still using it as a decision tool [19]. This is mainly due to its simple and intuitive application and because the ARIA and ASGC-RA classifications are not viewed as being superior measures for many rural populations [17,20]. All three classifications are deeply ingrained within Australian rural health policy, even though none were originally designed or intended for use as resource allocation decision tools.

**Table 1:** Summary of strengths and weaknesses of the RRMA, ARIA and ASGC Remoteness classifications

Classification	Strengths	Weaknesses
**RRMA**	• RRMA is a simple tool to apply both for research and administration purposes, including the allocation of health resources.	• The restriction to SLA boundaries, resulting in large, heterogeneous areas being equally classified.
	• Due to the strong influence of population size, RRMA often equally classifies towns of similar size (intuitive).	• The use of straight-line distances and SLA centroids, which can result in highly imprecise measures.
	• The use of three zones (metropolitan, rural and remote) is reasonably logical.	• The use of population density is meaningless because of the varying size and nature of SLA boundaries.
	• RRMA is preferred by many national organisations over ASGC Remoteness	• RRMA has never been updated and still uses 1991 population counts.

**ARIA**	• The flexibility to measure remoteness at any geographic boundary level by using a one kilometre grid.	• Only measures geographical remoteness, giving many examples of highly dissimilar towns having the same classification (e.g. Port Macquarie and Gundagai).
	• The additional precision from using road distances and service town locations, rather than straight line distances and SLA centroids.	• The separation of the five remoteness categories is somewhat subjective.
	• The clearer conceptualisation of measuring only geographical remoteness of localities (e.g. not muddied by also measuring density).	• Penalises smaller, more densely populated states (e.g. over 75% of rural Victoria's population is defined as 'highly accessible'.
		• Use of the category label 'accessible' and the term 'accessibility' within its name (it is not a measure of access)

**ASGC-RA**	• All points listed under ARIA, plus:	• All points listed under ARIA (except the last point), plus:
	• More refined methodology (additional service centre category, better separation of major cities)	• Extreme heterogeneity within some areas, especially Inner Regional and sometimes Outer Regional
	• A change of labels including the use of 'regional' rather than 'accessible'	
	• Updated by ABS as part of the ASGC	

## The significance of geographical classifications for rural health policy

In Australia and internationally, the supply of health care practitioners is problematic in many rural areas. Rural populations generally experience decreased accessibility and diminished availability of health care services, particularly as distance from capital or major cities increases and local population size decreases. This occurs most notably in the case of GPs because of their critical role within the health care system [21,22], but also for other important services including those provided by dentists, pharmacists and allied health professionals [19,23,24]. Recruitment and retention difficulties of the rural and remote health workforce stem from many associated factors, including practice characteristics and professional support, personal/family lifestyle issues, and geographical/community factors [25]. In response, over the last twenty years the Australian Government has provided additional incentives and resources to rural and remote areas characterised as difficult to recruit to or retain services within. At last count (mid - 2009), the Department of Health and Ageing manages approximately 66 current programs along with a number of additional state-based programs, largely because mainstream programs do not adequately meet the needs of practitioners in rural and remote communities [26]. In order to target the distribution of these limited resources, some variant of the RRMA, ARIA or ASGC-RA classifications have frequently been used as the basis for differentiating both entitlement to, and nature of, financial and support incentives. For example, the Rural Retention Program uses the GPARIA classification (a variant of ARIA, which measures both population remoteness and GP professional isolation), while rural loadings which range from 15% to 50% in the Practice Incentives Program (PIP) are based on RRMA categories.

A critical question is whether these classifications are the most appropriate bases for the distribution of these important but limited resources. Given that there is no 'natural' rural urban classification, it follows that decisions made about where you draw the boundary differentiating 'urban' from 'rural' or 'rural' from 'remote' directly affects the eligibility and amount that different rural communities receive and consequently how well the problem of workforce shortages in rural areas is addressed. As we see in Table 1, all classifications have weaknesses. The Australian Government has recognised, to some degree, the inappropriateness of currently used classifications recognised for rural health policy decisions [27,28], though their recent response of selecting ASGC-RA highlights the lack of any explicit rationale for their adoption of what is arguably a sub-optimal solution.

## What determines a satisfactory solution?

To date, there appears to have been a desire by policy-makers and others for a single all-purpose classification to guide the distribution of health care resources to rural communities, without significant debate about whether the defining variable is the degree of 'rurality' or 'remoteness' or some other aspect of accessibility, disadvantage or contextual factor that underpins the problems associated with health care provision in these eligible areas. Numerous authors have debated "what is rural" and sought definitions based on characteristics such as low population density and small population centres, isolated populations and large distances, as well as observed environmental, agricultural and other economic activities [29-33]. In reality, the related concepts of rurality and remoteness are multi-faceted, thus precluding agreement on one universally accepted classification [8,31,34]. Nonetheless, governments continue to seek some agreed objective measure or classification on which to base their resource allocation decisions. This search is not limited to Australia, with a number of alternate classifications in existence [35-42], but generally these too only capture similar elements of rurality and so they offer no significant design alternative to the Australian classifications.

Table 2 provides a summary of important characteristics and related required decisions associated with any geographical classification. The first and most important distinction is to be clear about its purpose; that is, what is the classification designed to measure. For example, the ASGC-RA classification was unambiguously designed to measure geographical remoteness of populations. On the other hand, the RRMA classification captures some elements of 'rurality' including population size and density. Within each classification method, subjective decisions are required that determine its outcomes and the consequent degree of acceptance by users. These decision points include the choice of algorithm, the number of groupings and how they are determined, as well as the size of spatial units. The RRMA classification, despite its inherent weaknesses, is still preferred by many groups over the ASGC-RA classification [43-47], chiefly because of its ability to discriminate between areas at a finer geographical scale, thereby giving somewhat more homogenous groupings. Rather than continue to search for a single solution that suits all applications, it is more appropriate to develop classifications closely aligned with a specific defined purpose. A number of examples illustrate what can be achieved:

**Table 2:** Summary of decisions required regarding important characteristics of geographical classifications

Important characteristics	Decisions required - sources of subjectivity
Be clear on specific objectives and purpose of the classification as this determines what is being measured	Is it remoteness, isolation, access, disadvantage, rurality or something else? If it is an access classification, then what aspect of access is being measured, and in relation to what service - (e.g. GPs as a measure of primary care)

The choice of algorithm or procedure for grouping similar clusters matters	Accessibility can be measured by distance to nearest service, service provider to population ratios, or increasingly sophisticated methods such as floating catchments and distance-decay

The criteria and cut-off points underpinning groups matters	How many groups do you want? At what point do you differentiate between groups? (e.g. Is the decision based on minimising within-group and maximising between- group variance, or is the number arbitrarily defined by convenience for the end-user?)

The choice of spatial units matters	RRMA is often criticised for its use of Statistical Local Areas (which can be large in rural areas), but the more extreme use of 1 km grids such as ASGC-RA is typically not an option for most data required.

i. The Griffith Service Access Frame (GSAF) is one classification that measures access disadvantage in relation to education services, and is intentionally designed as a tool for resource allocation [48,49]. The GSAF is characterised by measuring access only to the nearest service option, and has been adopted by many Australian states in the distribution of rural (education) resources. Such an option may be an appropriate method for measuring access to hospital and specialist care services in the field of health.

ii. More recently, McGrail's new index of rural access, tested in Victoria, has been developed as a more appropriate measure of access to primary care services in rural areas [50,51]. This index is specifically designed to include the key elements of access to GPs (availability, proximity, mobility and health needs), utilise more appropriate advanced accessibility methods (modified two-step floating catchment areas [52,53]) and use the smallest feasible geographical units (collection districts).

iii. The GPARIA classification, a modified version of the ARIA classification, was specifically developed for the purpose of distributing Rural Retention Program grants to GPs working in rural and remote communities. GPARIA measures both remoteness and isolation by incorporating proximity to nearby GPs of both the population and GPs in its construction. How well this classification adequately differentiates all aspects of factors affecting retention decisions across rural and remote communities is a moot point.

iv. The District of Workforce Shortage (DWS) status is a simple yes or no stratification for all Statistical Local Areas (SLAs), which has been regularly updated every quarter for over 10 years using Medicare data [54]. An area's DWS status reflects whether the ratio between population size and the number of services provided within an SLA is below the national average. It should be noted, however, that its value is questionable because population-provider ratios are a poor measure of access, particularly for 'small' rural areas [52,55] and its dichotomous definition does not allow small areal variations to be detected. However, new methods such as McGrail's index of access can improve its application.

In addition to forming the basis for resource allocation decisions, geographical classifications are often used as statistical tools to guide rural health research, and, in particular, the presentation of results such as health outcome measures in evaluating the effectiveness and quality of health care [56,57], or through measuring service utilisation rates as an indicator of need for services [22]. A few other examples include the association between cancer survival rates and ARIA [58], the association between primary care management of chronic heart failure and RRMA [59], the association between Attention Deficit Hyperactivity Disorder (ADHD) treatment and ASGC-RA [60], or the association between mental health status and RRMA [61] or ARIA [62]. Measures of the extent to which services and interventions are resulting in improvements in the health status of rural Australians are contingent upon how rural is defined.

The use of inappropriate classifications can serve to mask or average-out important health inequities that characterise rural communities. Many authors have failed to fully appreciate the significance of rural delimitation. Simply bundling together places of diversity (heterogeneous) into convenient (presumed homogenous) categories often obscures the inherent variations within rural areas [63] and seriously affects the resultant pattern of health status and differentiation [64]. Many possible covariates, such as differing demographics, socio-economic status, access to health services and health behaviour, are frequently not included within statistical reports that are broken down by geography, despite their possible influence on the extent to which apparent associations with rurality are significant. In short, while significant associations between geographical classifications and various health and health service outcomes are interesting, they often conceal the true effect within rural populations.

## Conclusion

Geographical classifications are a significant part of rural health workforce policy, as the government endeavours to improve or at least maintain the rural health workforce supply. However, these classifications are often inappropriately applied--arguably the case with the use of the RRMA, ARIA and ASGC-RA classifications in rural health policy in Australia. We argue that the recent 'official' selection of the ASGC-RA classification over RRMA or ARIA for resource allocation in Australia will see the continuation of inappropriate distribution of many rural health programs. This review has highlighted the improbability that one solution can be satisfactorily applied in all purposes. In relation to rural health funding distribution, programs are designed as incentives or compensation for working in areas characterised by aspects such as low levels of access, high isolation, high disadvantage, small population base and greater complexity of activity. Geographical classifications by themselves cannot capture all these aspects within a single measure. It may be more appropriate to develop a suite of classifications that are explicitly and unambiguously designed to meet the requirements of a specific purpose, such as McGrail's new index of rural access or the Griffith Service Access Frame. Any classification of rural communities must ensure that people experiencing similar characteristics and problems of location and environment fall within similar categories.

Arguably, this is a major weakness of the preferred ASGC-RA classification, because it often categorises highly dissimilar localities as being 'equal' (such as Bendigo-- large regional centre with a population of almost 100,000 and Rushworth--small rural town with a population of only 1,000). Clearly, clinicians based in locations such as these are likely to experience contrasting issues that require different support policies. It follows that any adjustment to the current formulae underpinning resource allocation will inevitably create 'winners' and 'losers'. However, failure to do so limits the effectiveness that these programs can have in achieving their objective of maintaining or improving the equitable provision of health care services to rural populations.

## Competing interests

The authors declare that they have no competing interests.

## Authors' contributions

MM conceived and developed the main themes of this manuscript. MM and JH collaborated in drafting and approving the final manuscript.
